# Medical AI across Data Regimes to Promote Proactive Health

**DOI:** 10.34133/hds.0495

**Published:** 2026-09-10

**Authors:** Pengfei Li, Jingyi Wu, Qianlin Zuo, Luxia Zhang, Qimin Zhan

**Affiliations:** ^1^ National Institute of Health Data Science, Peking University, Beijing 100191, China.; ^2^ School of Nursing, Peking University, Beijing 100191, China.; ^3^ Advanced Institute of Information Technology, Peking University, Hangzhou 311215, China.; ^4^ Center for Digital Health and Artificial Intelligence, Peking University First Hospital, Beijing 100034, China.; ^5^ PKU-WUHAN Institute for Artificial Intelligence,Wuhan 430074, China.; ^6^ Institute for Artificial Intelligence, Peking University, Beijing 100871, China.

## Abstract

Importance: Proactive health has emerged as a transformative paradigm in modern public health, shifting the traditional emphasis from episodic, reactive care toward continuous, anticipatory, and preventive health management. This shift is both timely and necessary in the context of rising chronic disease burdens, aging populations, and increasing demands on healthcare systems. Understanding how advances in medical data and artificial intelligence (AI) underpin this transition is essential for guiding future research and practice. Highlights: This review synthesizes the evolution of proactive health alongside major developments in medical data regimes and AI technologies. The progression of medical AI is characterized across 4 data regimes, including sparse-data, small-data, big-data, and the emerging full-data era characterized by large-scale multimodal data integration. In tandem, AI methodologies have advanced from early expert systems built on rule-based knowledge engineering to contemporary large multimodal models capable of unifying diverse healthcare data streams. These technological and data-centric breakthroughs are reshaping proactive health practices across multiple domains, from decentralized health monitoring, risk-adaptive screening, dynamic and responsive treatment, virtual rehabilitation and digital health intervention, to construction of integrated medical and health services. Conclusions: Despite these advancements, marked technical and ethical challenges remain, limiting the translation of proactive health into routine clinical practice. Addressing these issues will be essential for realizing the full potential of proactive health and enabling future public health systems that are more anticipatory, efficient, and equitable.

## Introduction

The generation, integration, and use of health data have undergone profound changes in recent decades, fundamentally reshaping how clinicians and health systems understand and respond to population health needs [1,2]. For much of the past century, decision-making took place within a sparse-data environment, where health information remained fragmented, delayed, and insufficiently standardized or accessible for large-scale computational use. The digitization of health records marked a major turning point. Electronic health records (EHRs), disease registries, and expanded reporting systems enabled the systematic capture of clinical information and its linkage across care settings. As interoperability improved, these previously isolated data began to support more comprehensive and timely insights for risk assessment, diagnosis, and treatment planning. This transition improved surveillance, strengthened policy evaluation, and enabled more responsive public health actions [3,4]. More recently, this trajectory has accelerated into a full-data era, in which large-scale medical, biological, environmental, and behavioral datasets together provide a multidimensional view of factors shaping health across the life course [5].

Clinical decision-making has evolved from a predominant reliance on expert experience toward a more integrated approach supported by empirical evidence and increasingly enriched by expanding health data. Artificial intelligence (AI), particularly machine learning (ML) and deep learning, now plays a central role in interpreting complex health data [6,7]. These tools enhance traditional approaches by improving diagnostic accuracy, refining risk assessment, and informing treatment planning. Emerging methods, such as explainable AI and causal inference [8,9], offer new opportunities to understand underlying mechanisms and generate insights that can support more transparent and trustworthy decision-making. Recent progress in large language models (LLMs) has further accelerated these developments [10,11]. Their ability to combine text, images, signals, and other multimodal data sources enables more comprehensive analyses than were previously possible. These models enhance communication tasks, from summarizing clinical information to supporting patient engagement, and improve the interaction between humans and digital systems. Together, these developments signal a data-driven public health landscape in which early detection, personalized interventions, and informed decision-making are increasingly achievable.

These changes are occurring alongside major demographic and societal shifts. Populations are aging, life expectancy is increasing, and chronic diseases now account for a growing share of the global disease burden [12,13]. Traditional passive models of health management, largely focused on episodic and reactive care, are not well suited to the continuous and complex needs of large, aging populations. At the same time, individuals are becoming more active participants in their own health. The widespread use of fitness trackers, mobile health applications, wearable sensors, and digital health interventions (DHIs) allows people to monitor daily behaviors and physiological measures, prompting earlier engagement with health risks [14]. Together, these trends point to a growing emphasis on proactive health management that aims to strengthen resilience, prevent disease, and support well-being across the life course. In the literature, proactive health is broadly defined as the set of social activities centered on maintaining and improving health, including controlling risk factors at their source, generating health value throughout the process, and actively responding to population-level health risks [15]. This shift reorients the focus from treating illness to maintaining continuous health, from disease-centered to health-centered care, and from patient-centered to people-centered approaches. Advances in big data and digital technologies further reinforce this transformation by creating a robust foundation for integrating proactive health management into routine practice, paving the way for more sustainable, person-centered, and prevention-oriented care.

In this review, we summarized the progression of medical AI through 4 data eras, from sparse-data to full-data, providing a concise overview of how these technological and data-centric breakthroughs are reshaping proactive health practices across multiple domains. We also discussed persistent challenges that continue to hinder the effective implementation of these technologies in clinical and public health settings.

## Data Regimes in Medical AI: From Sparse-Data to Full-Data

**Fig. 1.**
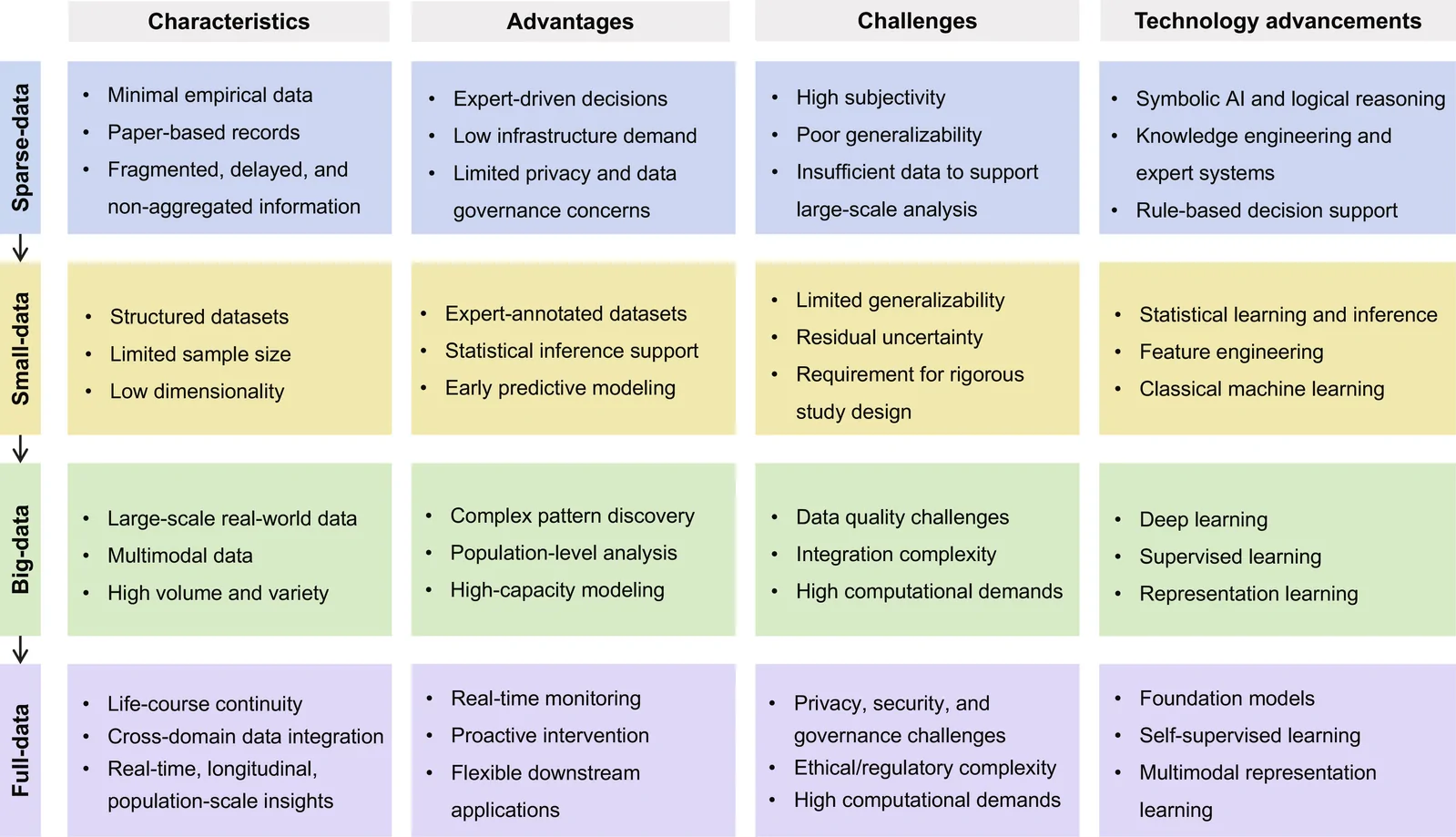
Overview of technology advancements across data regimes.

The evolution of medical AI reflects not only increasing data availability but also a sequence of responses to fundamental constraints in how health data can support clinical decision-making. Transitions between data regimes have been driven by the need to overcome specific bottlenecks, ranging from the limited availability of usable data, to insufficient representativeness, to challenges in scalability and integration (Fig. 1).

The sparse-data era (approximately 1950s to mid-1980s) emerged under conditions in which biomedical data, although present, were not structured or accessible for computational use. Fragmented infrastructures, lack of digitization, and limited computational capacity constrained the use of empirical data, necessitating reliance on formalized knowledge. Consequently, logic-based reasoning and expert systems dominated, representing a practical response to data inaccessibility rather than a purely theoretical preference. Systems such as MYCIN demonstrated that codified expertise could support clinical decision-making; however, their dependence on manually constructed rules limited scalability and adaptability. The shift away from this paradigm was therefore driven by the need for models capable of learning directly from data.

The small-data era (approximately mid-1980s to late 2000s) arose with the increasing availability of structured datasets from clinical studies and biomedical research. This period saw the expansion of curated biomedical databases such as GenBank, the launch and landmark initiatives like the Human Genome Project, alongside the formalization of statistical learning frameworks and the early development of ML methods. The relatively low dimensionality and restricted population coverage of small datasets often failed to capture the heterogeneity of real-world clinical and public health settings, limiting the applicability of findings to broader clinical or public health contexts. The central limitation was not merely data quantity, but the inability of curated datasets to capture the complexity and variability of health and disease, motivating the transition toward larger and more diverse data sources.

The big-data era (approximately 2010 to late 2010s) was driven by the recognition that model performance depends critically on the scale, diversity, and granularity of data. The widespread adoption of EHRs, supported in part by policies such as the Health Information Technology for Economic and Clinical Health Act, along with advances in medical imaging, and the growing use of biosensors and mobile health applications produced extensive datasets that capture diverse aspects of health and behavior. These multimodal data streams reflect real-world conditions at unprecedented scale and detail. Deep learning emerged as a response to the limitations of feature-engineered models, offering the ability to learn representations directly from high-dimensional data. While these developments addressed issues of scale and representation, they also introduced new challenges, including data heterogeneity, integration complexity, and dependence on labeled datasets, thereby motivating the next transition.

We are now advancing into a full-data era (approximately late 2010s to present), characterized by drawing knowledge from all available information across the healthcare system and the wider digital environment. This transition is driven by 3 key factors: the predominance of unlabeled data, necessitating self-supervised learning; the need for generalizable models that operate across tasks and domains; and the demand for continuous, real-time decision support. Health data are now generated continuously throughout the life course, spanning clinical encounters, daily behaviors, environmental exposures, and social contexts. Increasingly, non-health data sources are also being incorporated, such as food purchasing patterns, voice and speech features, and sentiment signals from digital interactions [16]. Such full-data environments enable real-time, longitudinal, and population-scale coverage that captures the complexity of health and disease more comprehensively than before. The millstones included transformer architectures and foundation models, enabling the extraction of general representations from large, unlabeled, and multimodal data, substantially enhancing model flexibility and performance on complex tasks while reducing dependence on manually curated training data. Full-data integration strengthens monitoring, supports early detection of emerging risks, and enables more precise and proactive interventions.

## Technology Advancements across Data Regimes

### Expert systems in sparse-data settings

In the sparse-data setting, decision-making depended almost entirely on domain knowledge rather than statistical induction. AI systems of this period, typically expert systems and rule-based clinical decision support tools [17], were built on logic, symbolic reasoning, and human-encoded expertise. Their defining characteristic was explicit knowledge representation, and their decision processes were fully transparent, understandable to clinicians, and well aligned with regulatory and safety expectations. In the absence of empirical examples, developers relied on structured knowledge representation, modular rule hierarchies, and systematic consistency checking to ensure validity and coherence.

However, expert systems’ reliance on manually constructed rules made them costly to build, difficult to scale, and burdensome to maintain as medical knowledge evolved [17]. Rule sets could become inconsistent or outdated, and incorporating new clinical evidence required extensive expert involvement. Their inability to learn from data and adapt to evolving knowledge limited their applicability in complex and uncertain environments, motivating the transition to data-driven approaches.

### ML in small-data settings

In small-data settings, statistical models and classical ML methods are typically used to extract patterns, with careful feature engineering and uncertainty modeling. Widely used methods include linear and logistic regression, support vector machines, random forests, early neural networks, ensemble learning methods, and so on [2]. Hidden Markov models and n-gram language models supported early advances in pattern recognition, speech processing, and natural language tasks [18,19]. These data-driven models can provide quantitative evidence of the patterns and relationships underlying risk factors and health outcomes, supporting more personalized risk assessment across heterogeneous populations [20]. Many of these ML models retain a degree of interpretability through feature importance analyses or decision-tree structures to enable transparent clinical decision support [2,21,22].

However, these ML models often struggle to handle high-dimension data and are often dominated by high variance, making overfitting a persistent concern. High ratios of features to samples, heterogeneous measurements, and limited external validity or generalizability are common challenges. The transition to deep learning was therefore driven by the need to better accommodate high-dimensional data and to reduce reliance on manual feature engineering.

### Deep learning in big-data settings

Recent growth in computational power and the availability of massive multimodal datasets have driven a shift toward big-data-based deep learning in healthcare research. Composed of multiple layers of representations, deep learning can learn complex patterns within and across data modalities while handling high dimensionality and intricate interactions, thereby reducing reliance on manual feature engineering and variable selection [23]. These models are well suited to multimodal data, supporting advanced interpretation of medical images, analysis of continuous physiological signals, and information extraction from clinical text. By integrating these diverse data sources, such methods improve the ability to reflect the multifactorial nature of disease and to construct more comprehensive representations of patient health. As a result, they can strengthen diagnostic support and improve prediction of clinical outcomes, often exceeding the performance of classical ML approaches [23]. With increasing sample sizes, these models also show greater scalability and adaptability across populations and care settings. Major advances have been reported in computer vision [24], natural language processing [25], and reinforcement learning (RL) [26].

Deep learning models are computationally intensive and require substantial infrastructure for training, validation, and deployment. Such demands can restrict use in low-resource environments and raise concerns about long-term sustainability. This lack of transparency and interpretability may reduce clinician confidence and complicate regulatory review and real-world implementation.

### Large multimodal model in full-data settings

Rather than relying on narrowly selected datasets, AI systems developed under a full-data perspective envision the integration of all available and continuously generated health data across the life course, to support more timely, personalized, and context-aware health management throughout prevention, care, and follow-up. This shift has enabled 2 influential paradigms: foundation models and generative AI. Foundation models are large, general-purpose systems trained on extensive datasets and adapted to a wide range of downstream tasks [10]. Generative AI focuses on learning data patterns to produce new content, such as text, images, or structured outputs. Together, these paradigms extend AI beyond task-specific applications toward more flexible systems that learn shared representations across domains [11]. Most foundation and generative models are built on the Transformer architecture, which is characterized by a self-attention mechanism that allows models to weight the relevance of different input elements and capture long-range relationships and complex dependencies. A defining feature of these models is their reliance on weakly supervised or unsupervised pretraining on large-scale data, followed by task-specific fine-tuning, distinguishing them from earlier generations of ML approaches. This architecture underlies LLMs and large multimodal models (LMMs), which represent a recent stage in AI development [27]. By integrating large-scale medical knowledge with advanced reasoning capabilities, these models are beginning to reshape precision care and patient interaction. They facilitate more natural communication between patients and digital systems, assist clinicians in synthesizing complex clinical information, and enable cross-modal analyses. Their capacity to adapt to new tasks with limited additional data further enhances their potential utility in dynamic healthcare environments.

Advances in LLMs and LMMs have also catalyzed the development of medical AI agents, autonomous computational systems that combine 4 interdependent functional modules: planning, which supports reasoning and decision-making; action, which enables task execution through multiple interfaces; reflection, which involves multimodal sensing, interpretation, and feedback; and memory, which allows relevant contextual information to be stored and retrieved over time [28,29]. By integrating these capabilities, medical AI agents can maintain continuity across clinical interactions, incorporate knowledge from prior experience, and adapt their responses as patient conditions and care contexts evolve. This architecture provides a foundation for more adaptive, longitudinal, and context-aware support across healthcare settings. The full-data era amplifies the strengths of medical AI agents by enabling comprehensive perception, continuous learning, and proactive decision-making, positioning them as key enablers of precision, preventive, and value-based healthcare.

## Changing Practice for Proactive Health

**Fig. 2:**
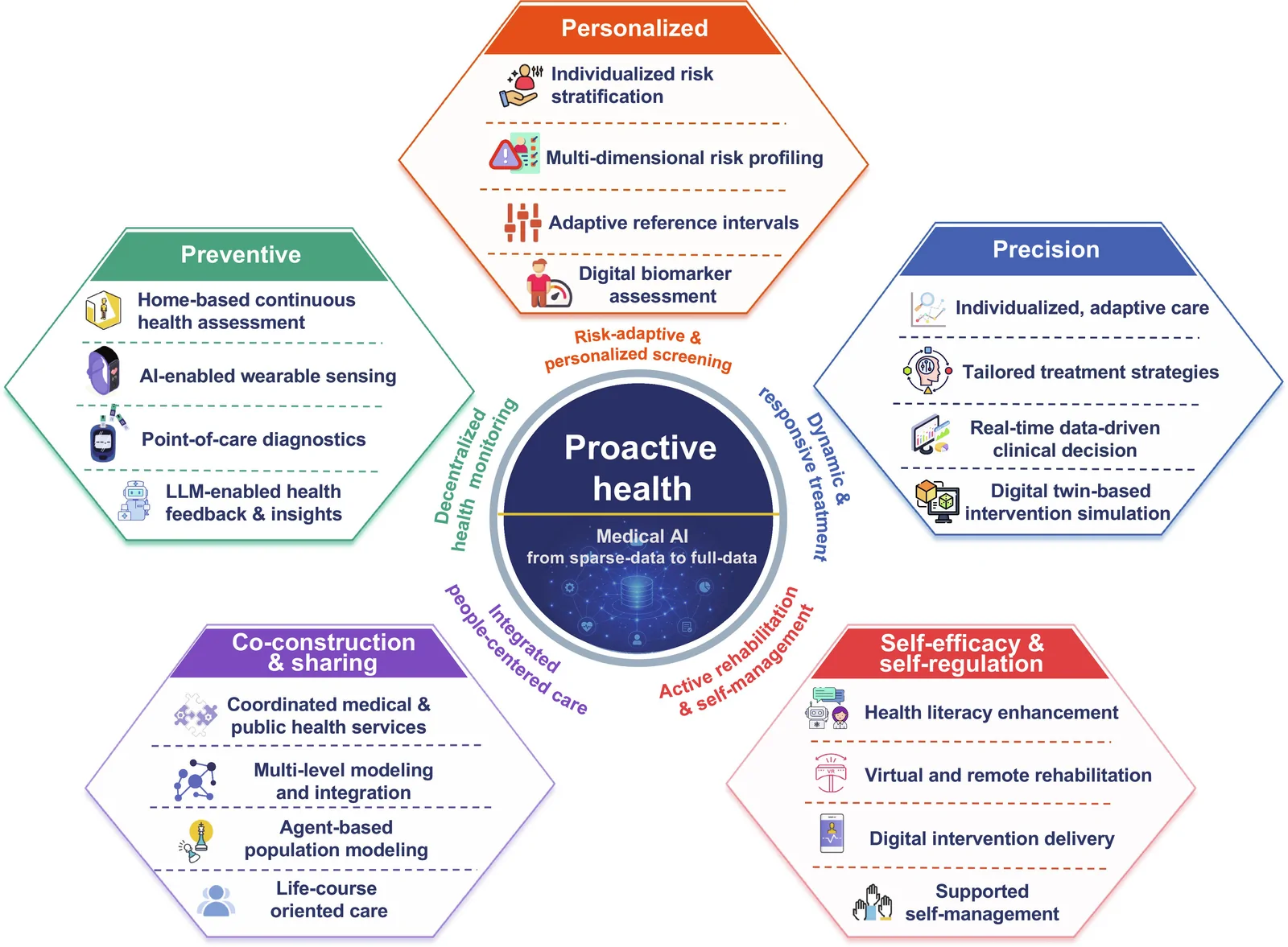
The landscape of proactive health driven by medical AI.

State-of-the-art advances in AI are signaling a shift toward a new paradigm of health management, shifting away from predominantly reactive, hospital-centered models toward proactive, personalized, and accessible health management (Fig. 2). Within this paradigm, individuals gain more continuous and fine-grained insight into their health status, enabling active self-management through timely and tailored feedback. Earlier identification of disease risk and timely intervention at preclinical or early stages can improve prognosis, reduce reliance on acute care resources, and ultimately enhance health outcomes. Meanwhile, AI-enabled tools allow traditional treatment and management strategies to become more dynamic and responsive to patients’ real-time health status. DHIs, in particular, are poised to fundamentally reshape rehabilitation and chronic disease management by enhancing self-efficacy, self-regulation, health literacy, and sustained engagement in care. More broadly, AI-powered systems support the emergence of integrated medical and public health services that are oriented toward the entire population and span the full life course. By coordinating interventions related to diet, physical activity, psychological well-being, environmental exposures, and social and cultural contexts, these systems have the potential to connect prevention, diagnosis, treatment, rehabilitation, nursing, and long-term care into a cohesive and continuous health service framework [15].

Importantly, this transformation is not driven solely by big-data or full-data paradigms. Methods developed in the sparse-data and small-data eras remain indispensable, particularly in settings where high-quality, large-scale data are unavailable or where interpretability, robustness, and causal reasoning are required. Rule-based systems and domain knowledge continue to support clinical decision-making in well-understood scenarios, while statistical learning approaches are essential for structured, moderate-scale datasets commonly encountered in clinical practice. These paradigms also play a critical role in ensuring model transparency, supporting hypothesis-driven analysis, and complementing data-intensive approaches when data are incomplete, biased, or difficult to access.

### Decentralized health monitoring

In sparse- and small-data settings, health monitoring was inherently episodic, relying on clinic visits and periodic testing. Data were collected infrequently and often retrospectively, limiting the ability to detect early changes or monitor disease trajectories in real time. Consequently, they are poorly suited to meet the increasingly complex and sustained healthcare needs of a rapidly aging population. In the big-data and full-data era, health monitoring shifts from passive data collection to an active, adaptive process that continuously informs decision-making. Emerging AI-enabled wearable sensors and point-of-care testing (POCT) devices have demonstrated the ability to provide real-time monitoring of physical health status and health-related behaviors while also supporting routine surveillance of disease-specific indicators among high-risk populations [30,31]. Commonly used wearable devices provide continuous measurements of fundamental physiological parameters, including heart rate, respiratory rate, body temperature, electrocardiographic signals, blood pressure, and oxygen saturation, which enable trigger early warnings of adverse events and clinical deterioration, such as the onset or progression of cardiovascular, neurological, and pulmonary diseases [32–34].

In parallel, POCT devices in research and early clinical settings have expanded the scope of decentralized health assessment. Examples include deep learning-assisted smartphone-based assay for clinical evaluation of COVID-19 [35], computer vision-based approaches for kidney health monitoring [36,37], and ML-enabled systems for tracking diabetes-related parameters [38]. Moreover, advances in LLMs have enabled their integration into wearable sensors and POCT tools, where natural language understanding capabilities can assist patients in interpreting health indicators and test results. By enabling timely feedback and personalized insights, these tools can strengthen individual engagement and motivation in health management and enhance health literacy, a core component of proactive health.

### Risk-adaptive and personalized screening

Early detection and personalized screening in full-data era are core components of proactive health, aiming to identify disease at preclinical or early stages by shifting from population-averaged screening strategies in sparse- and small-data settings to risk-adaptive, data-driven, and continuously updated approaches. AI systems enable multidimensional risk profiling through the integration of diverse data sources, including demographic characteristics, family history, comorbidities, genetic susceptibility, lifestyle behaviors, and environmental exposures, to estimate individual-level disease risk with greater precision. An illustrative example is Delphi-2M, a generative pretrained transformer (GPT)-based model of multi-disease progression trained on large-scale health record corpora [39]. This model can learn lifetime health trajectories and accurately predict the occurrence of more than 1,000 conditions over the subsequent 2 decades, including various cancers, diabetes, myocardial infarction, and mortality. By supporting stratified risk assessment and targeted screening strategies, such models facilitate more personalized and prevention-oriented approaches to healthcare.

Beyond long-term risk prediction, several AI systems have been developed to operationalize risk-stratified screening [40] and adaptive individual reference interval [41], which interpret test results relative to an individual’s historical baseline rather than population reference ranges. For example, a landmark study in the United Kingdom evaluated the cost-effectiveness and health benefits of integrating AI-based risk-stratified screening into the National Health Service (NHS) breast cancer screening program [40]. Using the Mirai AI model to tailor screening intervals according to each woman’s predicted risk, the study demonstrated that AI-guided screening could reduce unnecessary examinations while improving early detection, particularly among high-risk individuals. Researchers from Florida State University developed Lab-AI, an interactive system that offers personalized normal ranges using advanced LLM integrated with retrieval-augmented generation (RAG) from credible health sources [42]. LLM-based agents may further enhance proactive screening by improving patient understanding, engagement, and adherence to recommended follow-up and surveillance strategies [43].

For diseases that are difficult to diagnose and require longitudinal, multidimensional data to identify subtle behavioral or psychological changes, such as cancer and dementia, AI-enabled diagnostic tools have shown promise for detecting disease at preclinical or early stages [44]. For example, a meta-analysis comparing AI algorithms with clinicians of varying experience levels in skin cancer classification reported clinically comparable performance between AI systems (sensitivity 86.3%, specificity 78.4%) and expert dermatologists (sensitivity 84.2%, specificity 74.4%) [45]. Increasingly, research has focused on developing digital biomarkers for the early detection of Alzheimer’s disease using AI models, including limb-movement biomarkers, digital assessments administered via mobile or dedicated information and communication technology (ICT) devices, eye-movement biomarkers, and speech-based biomarkers [46]. Digital biomarkers are typically cost-effective, noninvasive, and easily repeatable, making them highly suitable for large-scale proactive health management.

### Dynamic and responsive treatment

There is substantial individual variability in treatment response across nearly all areas of medicine. Nevertheless, conventional treatment strategies in small-data settings largely follow a one-size-fits-all paradigm, relying on average treatment effects derived from clinical trials and guideline recommendations. Such approaches often overlook the pronounced heterogeneity in individual responses. With the emergence of the big-data era, AI systems enable the development of dynamic, individualized treatment strategies that integrate multidimensional risk exposures and comprehensive health information. By tailoring therapeutic decisions to each patient’s characteristics and evolving health status, AI-driven systems have the potential to maximize clinical benefit while improving the efficiency, safety, and cost-effectiveness of care at both the individual and population levels. RL models have been widely applied to support the customization of therapeutic strategies to individual patient profiles [47,48]. RL learns optimal decision policies through iterative interaction with a stochastic, evolving environment, continuously refining actions based on feedback signals. This paradigm is particularly well suited to healthcare settings that require sequential decision-making under uncertainty, such as designing adaptive therapeutic strategies in long-term patient management. Wang et al. [47] from Fudan University developed an RL-based dynamic insulin titration regimen (RL-DITR), in which a patient model was integrated to capture temporal trajectories of glucose dynamics and a policy network was designed to support sequential, multi-step treatment planning. RL-DITR demonstrated superior insulin titration optimization and improved inpatient glycemic control compared with junior and intermediate-level physicians. Choi et al. [48] developed RL-based models to guide individualized decisions on on-scene resuscitation duration in out-of-hospital cardiac arrest, showing that implementing individualized RL-derived policies could substantially improve survival to hospital discharge and rates of favorable neurological recovery.

Digital twins are also an increasing focus in the field of AI-driven individualized treatment. They refer to virtual representations of individuals designed to closely reflect the physiological and clinical characteristics of their real-world counterparts, and are iteratively updated over time [49]. Digital twins enable in silico experimentation, allowing clinicians and researchers to simulate potential interventions and evaluate the expected therapeutic responses of different treatment strategies, together with associated uncertainty, prior to clinical application. Many digital twin systems are constructed upon mechanistically grounded models that retain interpretability in biologically and clinically meaningful terms, thereby enabling clearer communication between clinicians and supporting explicit reasoning about underlying causal mechanisms. Although their deployment in routine clinical practice remains at an early stage, initial pilot studies have suggested promising utility [50–52]. Notably, a digital twin-based framework for prostate radiotherapy has been introduced that combines multimodal imaging data with machine learning-derived estimates of clinical target volume positioning uncertainties, thereby facilitating individualized radiotherapy plan generation [53].

Rapid changes in population health needs, disease patterns, and care delivery are challenging health systems designed around static information and delayed responses. Creating real-time adaptive clinical systems or medical agents oriented toward the principle of “real-time needs, real-time care” is therefore an emerging direction. These systems enable a shift from scheduled, uniform service delivery toward dynamic, responsive care pathways in which the timing, intensity, and content of care are continuously matched to individual needs. This involves the real-time data capture and streaming, dynamic risk detection and prediction, adaptive decision support, and continuous learning loops. Li et al. [54] proposed a portable, wireless platform for real-time, continuous, and adaptive bioelectronic wound management (a-Heal). This system integrates a wearable device capable of both wound imaging and therapeutic delivery with a machine learning-driven “physician” module. The ML component interprets wound images to determine the stage of healing and subsequently recommends appropriate therapeutic interventions, thereby enabling automated, data-driven treatment selection within a closed-loop care framework. The a-Heal system continuously evaluates wound healing progress, adjusts therapy in response to changing conditions, and communicates updates to clinicians through a graphical user interface that also allows manual intervention when necessary.

### Active rehabilitation and self-management

Rehabilitation and chronic disease management are crucial for improving quality of life and maintaining independence by helping people cope with long-term conditions like diabetes, cardiovascular disease, and dementia [55]. Typical strategies like physical therapy, self-management education, and functional training help prevent complications and enable fuller participation in daily life. However, in sparse- and small-data settings, these approaches have largely relied on episodic, clinic-centered models of care, limiting their ability to provide continuous and individualized support. AI-driven approaches are enhancing these efforts by strengthening health literacy, supporting self-care, and enabling more continuous forms of care that extend beyond traditional clinical settings [56]. This is better aligned with patients’ daily lives and long-term needs.

In rehabilitation, AI-enabled systems facilitate individualized exercise and recovery programs by monitoring movement patterns, tracking adherence, and assessing progress over time. These data allow rehabilitation plans to be dynamically adjusted to a person’s current functional status, with intensity modified as recovery evolves. Such approaches enable rehabilitation to continue safely and effectively outside formal care settings, improving continuity of care, reducing access barriers, and supporting sustained engagement during often lengthy recovery periods. Accordingly, AI-driven virtual rehabilitation (VRehab) or synonymously telerehabilitation, as well as robot-assisted rehabilitation have become increasingly prevalent in the post-event recovery of chronic conditions such as stroke and cognitive impairment [55,57]. Evidence suggests that home-based VRehab achieves health outcomes comparable to in-person rehabilitation and is superior to receiving no rehabilitation. Tang et al. [58] developed an AI-enabled multimodal smart home system designed to support continuous monitoring and functional assistance for individuals with post-stroke motor impairments. The platform integrates data streams from wearable sensors, ambient sensing devices, and adaptive automation technologies to enable longitudinal tracking of patient status and provide context-aware supportive interventions within the home environment. Moneta Health developed an automated therapy delivery system for cognitive rehabilitation, in which patients receive personalized, interactive cognitive exercises via a telephone-based voice agent [59].

In chronic disease management, DHIs are increasingly enhancing patient engagement and supporting core activities such as medication adherence, symptom monitoring, lifestyle modification, and remote follow-up [60]. Remote monitoring and virtual support can reduce the need for frequent in-person visits, thereby easing pressure on healthcare services while maintaining clinical oversight. For example, Maguire et al. [61] from the UK developed the Advanced Symptom Management System (ASyMS) to support real-time, continuous monitoring and management of chemotherapy-related toxicities. ASyMS enables patients to access tailored, evidence-based self-care advice at any time and has been associated with significant reductions in anxiety, as well as improvements in supportive care needs and self-efficacy. Dorsch et al. [62] developed a dynamic mobile DHI based on a just-in-time adaptive intervention (JITAI) framework to promote physical activity and lower-sodium food choices among patients with hypertension. By incorporating real-time data streams from wearable devices, JITAI delivers interventions at moments when behavior change is most likely to be effective.

### Integrated people-centered care

In sparse- and small-data settings, public demand for improved health has been centered primarily on medical treatment. However, as health awareness, health literacy, and healthcare consumerism continue to improve, greatest benefits are increasingly derived from integrated models of medical and health services that are population-oriented and span the entire life course [15,63]. This approach integrates interventions related to healthy diet, physical activity, physical and psychological well-being, environmental factors, and cultural contexts while encompassing the full continuum of care, from prevention and diagnosis to treatment, rehabilitation, nursing, and long-term health management. In this context, medical AI in the full-data era provides a foundation for a co-constructed and shared health system that integrates individual, industry, and social forces to promote health in all fields.

AI-based multi-level modeling enables the integration of individual-level health management with population surveillance and policy interventions, thereby amplifying the impact of prevention efforts [64,65]. By capturing dynamic interactions between individual behaviors and organizational or policy contexts, this approach supports more precise and effective prevention strategies. In particular, it facilitates the identification of individuals or communities most likely to benefit from specific interventions and enables assessment of how coordinated actions across sectors can generate greater health gains, thereby contributing to more coordinated, equitable, and sustainable public health systems. A commonly used approach in this context is agent-based modeling (ABM), which simulates and analyzes complex interventions within dynamic, real-world systems [66]. For example, Huang et al. [64] developed a systems science simulation framework for smoking cessation policy that combined microsimulation and ABM to incorporate detailed behavioral rules at the individual level (patient decision-making) while simultaneously representing multi-level interactions at the organizational level, including clinic policies and provider networks. ABM is also widely used in scenario simulation and policy evaluation. By comparing the potential impacts of different intervention options on health, cost, and equity, researchers can assess their long-term effectiveness and potential risks before policy implementation, thereby enhancing the foresight and scientific rigor of public decision-making. Lazebnik [67] proposed a data-driven hospitals staff and resources allocation model using ABM and deep RL, and improved patient treatment success and cost-effectiveness were achieved.

In recent years, many countries have implemented big data and digital tools-enabled integrated care interventions to facilitate data sharing and support healthcare delivery [68–70]. Integrated care aims to better align health and social care around individuals’ needs, thereby improving health outcomes and care experiences. For example, the ValueCare project in Europe developed and implemented outcome-based, integrated health and social care models for older adults with multimorbidity, leveraging information and communication technologies to enhance the sustainability of European health and social care systems [69]. In parallel, healthcare AI is increasingly being transformed from an assistive tool into a prevention-oriented, systemic infrastructure that supports more efficient and equitable integrated health governance.

## Challenges and Future Directions

**Fig. 3.**
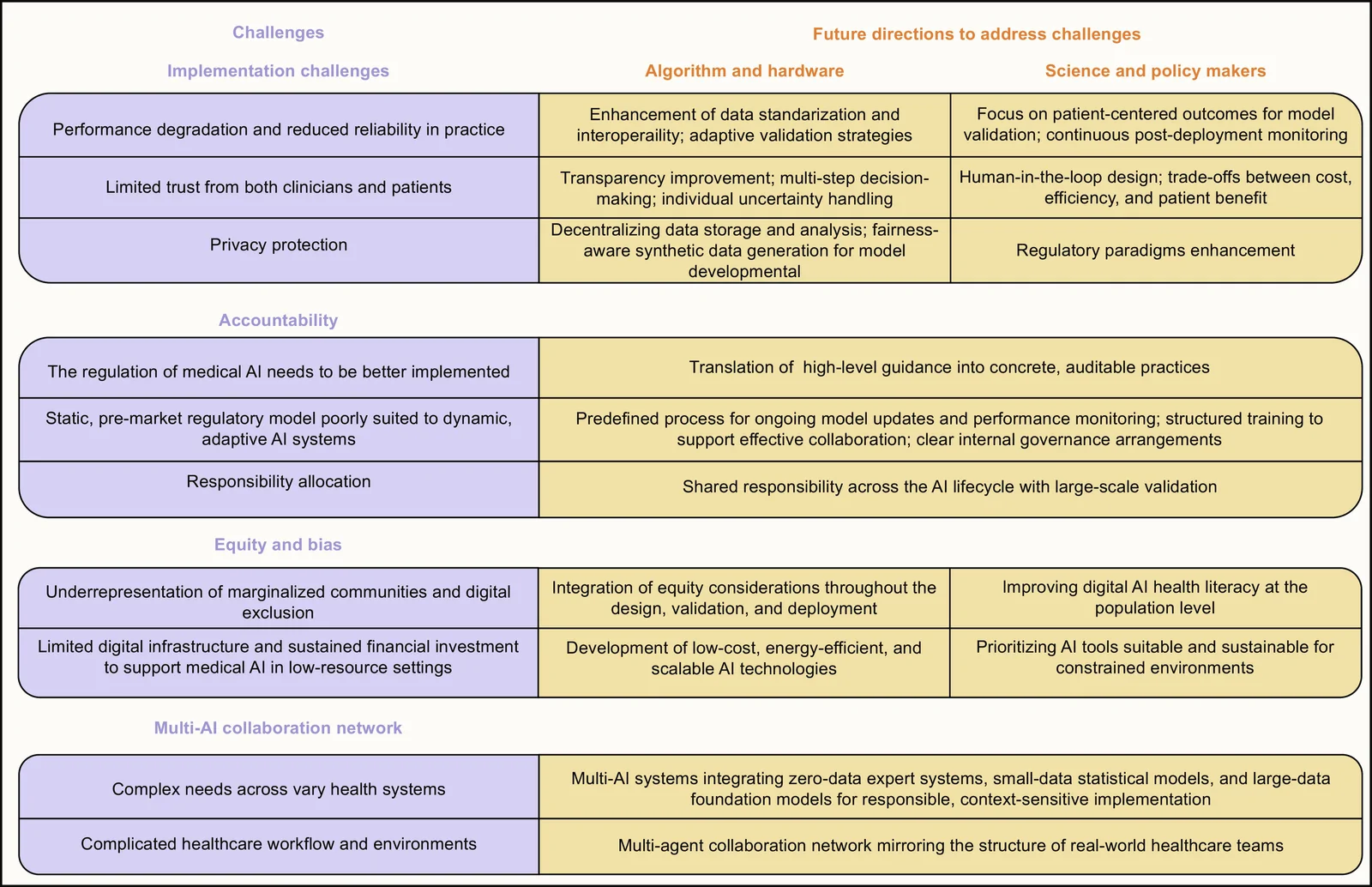
Challenges and future directions for medical AI.

By enabling earlier identification of health risks, continuous monitoring, and data-driven decision support, medical AI has been widely positioned as a key enabler of a shift from reactive care to proactive, preventive health paradigms. However, the rapid expansion of AI-enabled applications has also revealed a critical gap between technical capability and sustainable real-world impact. Despite promising performance in controlled settings, many medical AI systems struggle to achieve reliable integration, equitable benefit, and accountable use in routine practice (Fig. 3). These limitations are not confined to algorithmic design alone but emerge from complex interactions among clinical workflows, institutional infrastructures, regulatory frameworks, and broader social determinants of health. To move beyond proof-of-concept applications toward scalable and trustworthy proactive health systems, it is therefore essential to systematically examine the key challenges that constrain the implementation, governance, and societal alignment of medical AI.

### Implementation challenges

Despite sustained advances in model performance, a persistent gap remains between algorithmic promise under controlled conditions and the feasibility, safety, and practical utility required for successful clinical adoption, spanning early ML approaches to the latest LLMs. In real-world settings, effectiveness often falls short of expectations derived from retrospective analyses, with additional challenges including limited generalizability across clinical contexts and insufficient integration into routine workflows. Recent generations of LLMs and agentic systems introduce further complexities. Hallucination and unreliable reasoning remain concerns, particularly in high-stakes clinical environments, while outputs may vary across prompts, complicating reproducibility and validation. These systems also require substantial computational resources, which can limit scalability, and multi-agent architectures further increase system complexity, reducing transparency and making accountability more difficult to establish. In parallel, regulatory frameworks remain uncertain, especially regarding off-label use, and unanticipated challenges in human–AI interaction, such as overreliance on automated outputs and alert fatigue, continue to affect safe and effective deployment [11].

Trust from both clinicians and patients remains a major barrier to the real-world adoption of medical AI, especially for generative AI systems [71]. Although recent studies suggest progress in reducing hallucinations [72], substantial challenges remain. Ensuring trustworthy deployment requires not only enhanced regulation, transparency, and routine auditing but also advances in consent, privacy, and data security frameworks to support responsible data use. Emerging participatory approaches, including community-engaged data governance and co-design frameworks, offer promising models for building public trust by increasing transparency, accountability, and alignment with societal values. In addition, some recent models provide multi-step decision-making by exposing intermediate reasoning steps through structured prompting or built-in reasoning mechanisms to improve transparency [73]. Physician-in-the-loop approaches [74], where clinicians retain oversight and can review, correct, or override AI outputs, are emerging as a key strategy to strengthen accountability and safety. However, their effectiveness may be undermined by alert fatigue from excessive system prompts and automation bias, where users over-rely on AI and fail to challenge incorrect recommendations. In parallel, patient-in-the-loop approaches represent a complementary strategy [75]. Engaging patients helps ensure that AI tools reflect patient values, preferences, and real-world expectations, reducing the risk of misalignment between technological capabilities and clinical needs.

While many AI models achieve high average accuracy across populations, their predictions for individual patients or specific subgroups may be highly uncertain, which is an issue of particular importance in high-stakes clinical settings. Existing uncertainty estimation approaches often fail to capture this personalized uncertainty, limiting their usefulness for individual decision-making and contributing to patient- and clinician-level mistrust [76]. At the system level, increasingly sensitive monitoring and risk prediction tools may generate large volumes of alerts, placing strain on healthcare resources and potentially increasing unnecessary utilization and anxiety among the “worried well”. These dynamics raise important health economic challenges regarding how systems prioritize, triage, and respond to risk signals while balancing cost, efficiency, and patient benefit [77].

Stringent privacy requirements and high compliance costs have accelerated the use of synthetic data to augment training for large language and vision–language models. Although synthetic data can expand training datasets and mitigate privacy risks, it may also reinforce existing biases, underrepresent rare but clinically important cases, and introduce spurious associations. Overreliance on synthetic data can therefore compromise real-world performance and foster unwarranted confidence in AI systems, particularly in the absence of robust validation using real clinical data [78].

### Accountability

Despite its growing influence, the role of medical AI within existing regulatory frameworks remains poorly defined. This uncertainty not only complicates the assignment of responsibility but also amplifies ethical and legal risks associated with AI deployment. Several international initiatives have proposed high-level guidance, including the European Union’s principles for trustworthy AI [79], the U.S. National Institute of Standards and Technology risk management framework [80], and standards developed by the International Organization for Standardization [81]. While these frameworks provide important normative direction, they remain difficult to translate into concrete, auditable practices within real-world clinical settings [82,83].

These governance challenges are further intensified in the full-data era, where the development and deployment of AI systems increasingly depend on access to large-scale, often privately held datasets. Much of these data remain siloed across healthcare providers, technology companies, and commercial platforms, limiting interoperability and constraining broader public benefit. For policymakers, this creates a complex balancing problem: enabling data sharing and innovation while safeguarding patient privacy, protecting commercial interests, and ensuring equitable access to the benefits of AI.

Governance challenges are further intensified by the dynamic nature of medical AI systems. Many AI tools are distributed across institutions, adaptive over time, and capable of continuous learning, whereas most regulatory models are still built around static, pre-market approval of discrete products. This mismatch limits regulators’ ability to respond effectively to model updates, changes in clinical context, and deployment across diverse healthcare environments [84,85]. The U.S. Food and Drug Administration has proposed an approach for adaptive AI systems that involves approving not only an initial model but also a predefined process for ongoing updates and performance monitoring [86]. To support effective collaboration, healthcare professionals require structured training that clarifies both the capabilities and limitations of AI tools. In parallel, healthcare institutions must establish clear internal governance arrangements that define responsibilities for system oversight, performance monitoring, and error management. Beyond institutional and regulatory measures, governance models that emphasize shared responsibility across the AI lifecycle are increasingly recognized as essential [87–89].

### Equity and bias

A major concern is that the benefits of medical AI are not distributed evenly across population groups, with the potential to reinforce existing health inequities [90,91]. Marginalized communities are often underrepresented in training datasets, which can result in systematic bias in model performance by socioeconomic status and geographic location. At the same time, acceptance of emerging AI-enabled interventions, such as DHI- and LLM-based care, varies widely across communities. Differences in socioeconomic conditions, cultural values, digital literacy, and historical trust in healthcare systems influence who is willing or able to engage with AI-supported services [92]. This digital exclusion can amplify disparities in who benefits from AI-driven innovations [91,93]. Inclusiveness and equity have been identified by the World Health Organization (WHO) as core principles of AI governance [94]. The WHO emphasizes that AI systems should be trained, validated, and monitored in the populations for which they are intended, and should undergo re-evaluation or retraining when deployed in new settings or among different groups.

Effective deployment of medical AI often depends on reliable digital infrastructure, skilled personnel, and sustained financial investment. In settings where these resources are limited, AI tools may be unavailable, difficult to maintain, or poorly integrated into care delivery, widening the gap between well-resourced health systems and those with fewer technological and financial capacities [95]. Recently, the prevailing reliance on large, general-purpose medical AI models raises additional equity concerns related to energy consumption and resource efficiency. The development of alternative technical pathways that are low-cost, energy-efficient, and scalable has become an important strategy for promoting equitable access.

### Multi-AI collaboration network

The emergence of the full-data era not only increases the scale and diversity of health data but also creates demands that are difficult for any single AI paradigm to address alone. Healthcare decision-making increasingly requires the integration of heterogeneous data sources, continuous adaptation across contexts, and coordination among tasks involving prediction, reasoning, communication, and workflow management. These requirements have motivated the development of collaborative multi-AI systems that combine the complementary strengths of sparse-data expert systems, small-data statistical models, and large-data foundation models.

Although foundation models and LMMs trained on large-scale data have demonstrated strong performance across many healthcare tasks, they do not represent a universal solution. In practice, expert systems that rely on domain knowledge rather than data, as well as traditional statistical and ML models trained on carefully curated datasets, continue to play an essential role, particularly in settings where data privacy, interpretability, and accountability are paramount. The complementary strengths of these approaches have led to the emergence of collaborative multi-AI systems, in which different types of models work together to address complex healthcare challenges in a more effective and responsible manner [96,97]. For example, an LLM model may identify patients at potential risk using complex data streams, while a transparent statistical model or expert system subsequently guides clinical action in accordance with established guidelines. Such collaborative designs also enable a more gradual and context-sensitive pathway for AI adoption. Health systems vary widely in data availability, technical infrastructure, and regulatory readiness. A flexible framework that integrates sparse-data expert systems, small-data statistical models, and large-data foundation models allows institutions to adopt AI incrementally, aligned with local needs and constraints.

Within this broader collaborative paradigm, multi-agent collaboration networks are emerging as a key enabling architecture, where AI systems not only operate autonomously but also learn and adapt through interaction [28]. By coordinating task decomposition, allocating computational resources efficiently, and assigning clearly defined responsibilities, multi-agent systems can reduce the complexity of executing healthcare workflows and improve overall reliability [98]. Clinical care, public health surveillance, and health system management often involve interdependent steps requiring different forms of expertise and data. A multi-agent framework mirrors the structure of real-world healthcare teams, with agents responsible for tasks such as data preparation, risk assessment, guideline interpretation, and communication with clinicians or patients. However, increased autonomy and inter-agent interaction also introduce challenges, including greater system complexity, reduced transparency, and heightened risks related to security, privacy, and accountability. Recent discussions surrounding agentic AI frameworks such as OpenClaw have highlighted concerns that increasingly autonomous systems capable of coordinating tools, memory, and external actions may expand the attack surface for data leakage, unintended behaviors, and loss of human oversight. In healthcare settings, these issues may complicate oversight and responsibility attribution, underscoring the need for robust governance and human oversight in real-world deployment.

## Conclusion

In the full-data era, AI advancements like continuous health monitoring using wearable sensors and POCT devices, longitudinal risk profiling, dynamic and individualized treatment guided by RL, and VRehab may progressively transform healthcare from a reactive, hospital-centered model into a proactive, health-optimizing system with finely tailored levels of intervention. In the near term, the most achievable transformations are likely to occur in relatively well-defined and data-rich settings, such as remote monitoring for chronic disease management, AI-assisted risk stratification for cancer and cardiovascular disease screening, and adaptive DHIs for behavioral modification. These applications are already showing early evidence of feasibility and clinical utility, particularly when integrated into existing care pathways rather than deployed as fully autonomous systems.

However, substantial gaps remain between the broader vision of full-data proactive health and current clinical reality. Current AI systems continue to face important technical limitations, including hallucination, instability across contexts, limited interpretability, and high computational demands. Achieving reliable real-world implementation will therefore require rigorous clinical validation, continuous post-deployment monitoring, incorporation of patient-centered values into AI-driven decision-making, and strict human-in-the-loop accountability. In parallel, the development and dissemination of low-resource medical AI systems are essential to improving the equity of health benefits derived from information technologies. Healthcare providers require adequate training and ongoing support to appropriately integrate AI tools into clinical practice, ensuring that these technologies enhance care delivery rather than adding further burden to an already overstretched workforce. Researchers, clinicians, health systems, and policymakers should adopt an integrated and coordinated approach across diverse data settings to foster responsible human–AI partnerships and establish robust governance frameworks while anticipating the transition toward a full-data and foundation-model era with caution, scientific rigor, and a strong commitment to equity.
